# Markers of endothelial glycocalyx dysfunction in Clarkson disease

**DOI:** 10.1186/s12967-022-03587-1

**Published:** 2022-08-29

**Authors:** Zhihui Xie, Magne Børset, Kjell Svéen, Ole Wilhelm Bøe, Eunice C. Chan, Justin B. Lack, Katherine M. Hornick, Franco Verlicchi, A. Robin Eisch, Remo Melchio, Arkadiusz Z. Dudek, Kirk M. Druey

**Affiliations:** 1 Lung and Vascular Inflammation Section, Laboratory of Allergic Diseases, National, Institute of Allergy and Infectious Diseases/National Institutes of Health, (NIAID/NIH), 10 Center Drive, Room 11N238A, Bethesda, MD 20892 USA; 2 Department of Clinical and Molecular Medicine, Norwegian University of Science and Technology, Trondheim, Norway; 3 Department of Immunology and Transfusion Medicine, St. Olav’s University, Hospital, Trondheim, Norway; 4 NIAID Collaborative Bioinformatics Resource, NIAID/NIH, Health, Bethesda, MD 20892 USA; 5 Transfusion Medicine Faenza-Lugo, Transfusion Service Ravenna, Romagna Health Unit, Ravenna, Italy; 6 Department of Internal Medicine, Santa Croce E Carle’ Hospital, Via Michele Coppino 26, Cuneo, Italy; 7 HealthPartners Neuroscience Center, St. Paul, MN USA

**Keywords:** Capillary leak, Endothelium, Glyocalcyx

## Abstract

**Background:**

Clarkson disease (monoclonal gammopathy-associated idiopathic systemic capillary leak syndrome, ISCLS) is a rare idiopathic condition marked by transient, relapsing-remitting episodes of systemic microvascular hyper-permeability, which liberates plasma fluid and macromolecules into the peripheral tissues. This pathology manifests clinically as the abrupt onset of hypotensive shock, hemoconcentration, and hypoalbuminemia.

**Methods:**

We analysed endothelial glycocalyx (eGCX)-related markers in plasma from patients with ISCLS during acute disease flares and convalescence by ELISA and comprehensive proteomic profiling. We evaluated eGCX-related components and gene expression in cultured endothelial cells using RNA-sequencing, real-time PCR, and fluorescence staining.

**Results:**

Serum levels of eGCX-related core components including hyaluronic acid (HA) and the core proteoglycan soluble syndecan-1 (sCD138) were elevated at baseline and during acute ISCLS flares. Serial measurements demonstrated that sCD138 levels peaked during the recovery (post-leak) phase of the illness. Proteomic analysis of matched acute and convalescent ISCLS plasma revealed increased abundance of eGCX-related proteins, including glypicans, thrombospondin-1 (TSP-1), and eGCX-degrading enzymes in acute compared to remission plasma. Abundance of endothelial cell damage markers did not differ in acute and baseline plasma. Expression of several eGCX-related genes and surface carbohydrate content in endothelial cells from patients with ISCLS did not differ significantly from that observed in healthy control cells.

**Conclusions:**

eGCX dysfunction, but not endothelial injury, may contribute to clinical symptoms of acute ISCLS.

Serum levels of of eGCX components including sCD138 may be measured during acute episodes of ISCLS to monitor clinical status and therapeutic responses.

**Supplementary Information:**

The online version contains supplementary material available at 10.1186/s12967-022-03587-1.

## Background

ISCLS, first reported by Clarkson et al. in 1960 [1], is rare disease that leads to intermittent, but ultimately self-reversing episodes of severe plasma extravasation. [2, 3]. Although fewer than 500 cases have reported in the literature, ISCLS may be under-reported due to misdiagnosis [4]. In addition to hypotensive shock, complications of acute ISCLS include renal failure, venous and arterial thromboses, rhabdomyolysis, and compartment syndromes resulting from extensive edema of the extremities. The 5-year survival rate has been reported to be between 73–78%, although these estimates generally antedate the widespread use of disease-sparing prophylaxis with intravenous immunoglobulins (IVIG) [5].

ISCLS flares are frequently preceded by upper respiratory infections (URIs) or flu-like symptoms, suggesting a role for inflammation in disease pathogenesis [4]. Increased levels of inflammatory cytokines such as C-X-C chemokine motif 10 (CXCL10), TNFα, and IL-6, and mediators of endothelial permeability (vascular endothelial growth factor [VEGFA] and angiopoietin-2 [Angpt2]) have been detected in acute ISCLS plasma [6–8]. While > 85% of patients have a monoclonal gammopathy of undetermined significance (MGUS), typically of the IgG kappa isotype [2, 5], the function(s) of the ISCLS “paraprotein” in disease pathogenesis has not been established. Monthly prophylaxis with IVIG induces disease remission in most patients and increases survival [5, 9].

The eGCX, which is comprised of proteoglycans, glycosaminoglycan (GAG) chains, and glycoproteins, coats the surface of the vascular endothelium and serves essential functions in vascular barrier function [10]. Microvascular endothelial cells continually secrete eCGX components, which serve as a molecular filter by constraining firm attachment of cytokines [11]. eGCX also prevents microvascular thrombosis and limits leukocyte adhesion through interactions with plasma albumin, antithrombin-III (AT-III), and extracellular superoxide dismutase-3 (SOD3), among other proteins [12]. Inflammation disrupts the eGCX and promotes vascular permeability, leading to shedding of soluble eGCX components such as HA and CD138 into plasma [11, 12]. Analysis of eGCX components has been used to diagnose and monitor disease states; elevated levels of plasma HA may predict progression and severity of heart disease, diabetes, sepsis, trauma, and ischemia-reperfusion injury, among others [13].

Previously we detected elevated serum levels of sCD138 in a single patient with active ISCLS, which normalized during disease remission [14]. Here we present the results of long-term follow-up of this patient and analysis of eGCX components in a larger cohort of individuals with ISCLS (n = 25). Our results suggest that monitoring these elements may be useful to gauge disease activity and that restoration of eCGX function could be explored to prevent or ameliorate acute flares of ISCLS.

## Materials and methods

### Patients

Demographics of patients studied are shown in Table 1. Age-, sex-, and ethnicity-matched serum samples from anonymous donors were obtained from the NIH Blood Bank.

**Table 1:** Demographics of ISCLS subjects

Variable	ISCLS	Controls
N	25	24
Sex (%female)	32	45.8
Age (years)*	49 (8–76)	49.5 (22–80)
Ehtnicity (%non-Caucasian)	11.1	8.3

^*^At time of diagnosis (median, range)

### Cytokine analysis

Serum CD138, HA, and thrombomodulin were measured by ELISA (R&D Systems or Abcam). SOMAScan screening of ISCLS plasma was described previously [15].

### Blood outgrowth endothelial cells (BOECs)

Endothelial cells were cultured from whole blood samples as described previously [16].

### RNA-sequencing

RNA from BOECs was reverse transcribed to cDNA and libraries constructed using the IlluminaTruSeq DNA Library Preparation Kit. Samples were sequenced on the Ilumina platform (40 M reads/sample, Beckman-Coulter Genomics). Raw fastq files were trimmed for quality and adapter contamination using Cutadapt v1.18. Trimmed reads were mapped to the hg38 reference genome and Gencode GRCh38 v.39 transcriptome using STAR v2.7.6a [17] in two-pass mode. Gene-level expression quantification was performed using RSEM v.1.3.0. Genes not expressed at a level greater than one count per million (CPM) reads in at least three of the samples were excluded from further analysis. The gene-level read counts were normalized using the trimmed means of M-values (TMM) in edgeR [18] to adjust samples for differences in library size. Differential expression analysis was performed using the quasi-likelihood F-test with the generalized linear model (GLM) approach in edgeR. Significantly differentially expressed genes (DEGs) in ISCLS and control samples were defined as those with false discovery rate (FDR) < 0.1. Principal component analysis (PCA) was performed in edgeR ‘prcomp’ built in function in R v.1.4.3.

### Gene expression analysis

RNA was extracted from BOECs using the RNAeasy kit (Qiagen) and cDNA was generated using SuperScript reverse transcriptase mix (ThermoFisher). qPCR was performed using gene-specific TaqMan probes (ThermoFisher) according to the manufacturer’s guidelines. Catalogue numbers for probes used are as follows: *Hyal1* Hs00201046_m1; *Hpse* Hs00935036_m1.

### eGCX detection in vitro

BOECs were stained with fluorescein-conjugated Ulex Europaeus (Gorse) Agglutinin I (UEA I, ThermoFisher) (final concentration, 5 μg/mL for 30 min at room temperature) as described previously [19] and fluorescence was visualized using a Leica DMI4000 microscope. Values were quantified at 459 nm (emission) and 515 nm (excitation) in a plate reader. Cell viability was determined using PrestoBlue (ThermoFisher), and UEA-1 values were normalized by cell number.

### Statistics

Non-RNA Seq statistical analyses were performed using GraphPad Prism software. Non-parametric Mann–Whitney (two-group) or Kruskal-Wallis (multiple groups) were used for comparisons. Non-parametric Spearman coefficients were calculated using simple linear regression. *p* < 0.05 was considered statistically significant. For RNA Seq analysis, preranked gene set enrichment analysis (GSEA) was performed using the WebGestalt online tool with default parameters [20]. Enrichment databases included Gene Ontology (GO) category ‘cellular component, non-redundant’ and PANTHER pathway. Significant enrichments were those with an FDR q-value < 0.1.

## Results

### Case report

Pt. 1 is a 49-year-old woman, who was diagnosed with ISCLS in 2009 after she presented with syncope following an upper respiratory tract infection (URI). The details of her initial presentation and hospitalization have been described previously [14]. Briefly, she was found to be profoundly hypotensive (blood pressure [BP] 60/40 mm Hg) and tachycardic (heart rate 85–105 beats per min[bpm]). She was resuscitated with intravenous saline (~ 20 L), which led to the development of generalized peripheral edema and compartment syndromes in both lower extremities that required fasciotomies. Laboratory investigations confirmed the diagnosis of ISCLS including the findings of hemoconcentration (peak hematocrit 73%), hypoalbuminemia (nadir serum albumin 1.8 g/dL), and a monoclonal IgG-kappa paraprotein (01–0.2 g/dL).

Over the past thirteen years, the patient has been followed regularly while on prophylactic treatment with theophylline and terbutaline She has not had a relapse of ISCLS despite experiencing several URIs during this period. In October 2019, 6 months after voluntarily discontinuing treatment, she experienced a sudden, acute visual disturbance in both eyes. Previously she had undergone lens removal from both eyes due to cataracts with restoration of vision. Examination showed several new rifts in the retinae bilaterally, which required laser-mediated repair and resulted in a considerable permanent loss of vision. Laboratory markers did not reveal any systemic signs of ISCLS relapse including a normal hemoglobin (15.4 g/dL) and hematocrit (47%). Beginning in February 2020, prophylactic therapy with IVIG was initiated (1 g/kg body weight), but this was discontinued in August 2020 due to adverse side effects (headaches). In September 2020, prophylaxis with terbutaline (7.5 mg po bid) and theophylline (Theo-dur 300 mg po bid) was re-started, and symptoms of ISCLS or retinal rifts have not recurred.

### Humoral eGCX markers in ISCLS

**Fig. 1:**
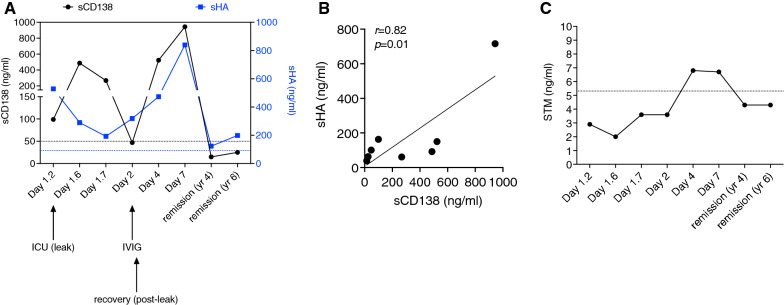
eGCX markers fluctuate over the course of an acute ISCLS flare. **A** Serum concentrations of hyaluronan (HA, blue) and sCD138 (black) during hospitalization (note: sCD138 data reproduced from [14]). **B** Correlation between sCD138 and sHA levels. **C** sTM levels over time

We monitored serum levels of eGCX components and endothelial-derived proteins in this patient over time. HA is a negatively charged GAG that is secreted on the endothelial surface and linked to the endothelial surface receptor CD44 in caveolae [13]. As previously reported [14], soluble CD138 (sCD138) increased in a biphasic pattern during the acute flare, rising above the normal range on day 1 of hospitalization followed by normalization and a second peak during the post-leak resolution phase on day 7 (Fig. 1A). By contrast, soluble serum HA levels were initially normal, followed by a more gradual rise that peaked on day 7. Overall, there was a significant correlation between sCD138 and HA levels (Fig. 1B). TM is an anticoagulant proteoglycan integral membrane protein on endothelial cells; increased soluble TM may indicate endothelial cell injury [21]. Serum TM was not elevated in this patient during the active leak phase of the ISCLS flare and was only minimally elevated during the resolution phase (Fig. 1C). Subsequently, serum levels of sCD138, HA, and TM remained within the normal reference values for 6 years after the start of the flare.

**Fig. 2:**
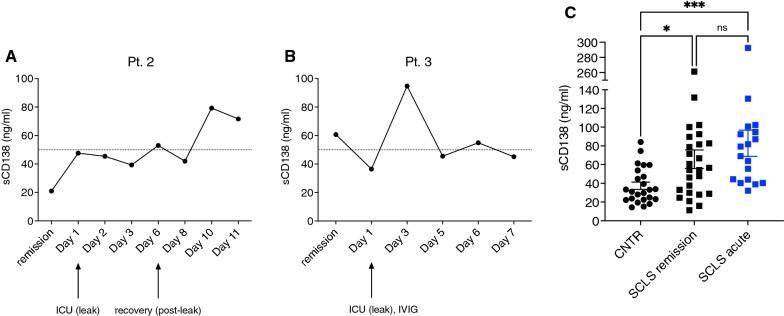
sCD138 levels in ISCLS. **A**, **B** sCD138 levels during acute hospitalization for acute ISCLS in two subjects. **C** sCD138 in patients with ISCLS during remission or acute phase of disease (mean ± S.E.M., *p = 0.03, ****p = 0.0004 vs. healthy controls, Kruskal Wallis ANOVA, Dunn’s multiple comparisons)

Based on our preliminary findings in this patient, we examined serial sCD138 levels in additional subjects. Pt. 2 is a 59-year-old man who experienced a severe ISCLS crisis in March 2009, as previously described [6]. Briefly, his hospital admission was characterized by protracted, pressor-dependent hypotension, hemoconcentration (initial Hgb level > 20 g/dL), and compartment syndromes, which were treated with fasciotomies. While sCD138 levels were normal during an asymptomatic interval 6 months prior to presentation, they increased slowly during his hospitalization, peaking on hospital day 10, during the recovery phase characterized by resolution of peripheral edema and diuresis (Fig. 2A).

Pt. 3 is a 48-year-old man who first presented in 2014 with hypotension (systolic blood pressure [BP] ~ 60 mmHg), hemoconcentration (Hgb/Hct 24 g/dL and 68%, respectively), hypoalbuminemia (nadir 2.6 g/dL), and IgG kappa MGUS. He was treated with norepinephrine and intravenous fluids; his course was complicated by anasarca, rhabdomyolysis (peak creatine phosphokinase 8900 U/L), and fevers resulting from a venous catheter infection. He recovered, and prophylaxis with oral theophylline (200 mg tid) was started. The patient remained asymptomatic until January 2015, when he presented to the emergency department (ED) with flu-like symptoms and edema in the upper extremities bilaterally. In the ED, he was found to be hypotensive (BP 80/40 mmHg) and tachycardic (heart rate 130 bpm). He was resuscitated with intravenous fluids and treated with IVIG (1 g/kg body weight) on the second day of admission to the ICU. sCD138 levels were normal upon presentation but increased, peaking on day 3 of hospitalization (Fig. 2B), at which time clinical recovery had begun as evidenced by stabilization of BP and gradual resolution of edema. He was discharged on day 11 of hospitalization. Prophylaxis with IVIG (1 g/kg/month) was begun, and he has not experienced further ISCLS flares.

**Fig. 3:**
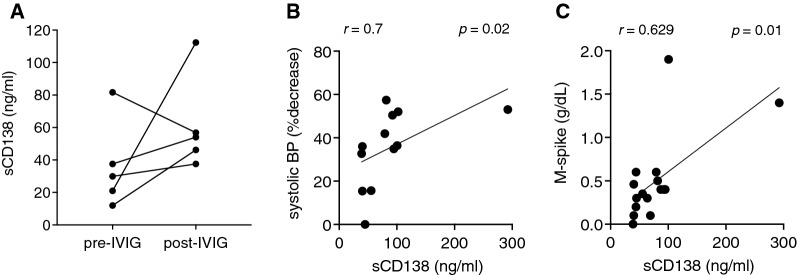
Correlation between sCD138 and clinical parameters. **A** sCD138 levels in patients pre- and post-IVIG prophylaxis. **B**, **C** Correlation between sCD138 and maximal decrease in systolic blood pressure from baseline (**B**) or serum paraprotein levels (**C**)

Finally, we examined sCD138 in additional subjects from our NIH cohort (n = 25), during both convalescent and acute intervals, and in healthy controls. Reference sCD138 levels vary with age and between published studies. We found that the mean (± S.EM.) levels in healthy controls were 36.69 ± 3.66 ng/mL (n = 24), which were within the range of values reported previously in both healthy children (2.8 ng/mL) [22] and adults > 18 years of age (19.3–42.2 ng/mL) [23–25] (Fig. 2C). Unexpectedly, sCD138 levels were significantly increased in both remission and acute ISCLS sera compared to controls (65.7 ± 9.9 and 80.8 ± 13 ng/mL, respectively (n = 19–26/group). We did not observe a consistent pattern of change in sCD138 levels in patients after initiation of IVIG prophylaxis (Fig. 3A). However, there was a significant correlation between acute sCD138 levels and clinical episode severity, as reflected by the maximum decrease in systolic BP (Fig. 3B). Baseline levels of sCD138 were also significantly correlated with serum paraprotein quantities (Fig. 3C). We did not find any significant correlations between sCD138 and ISCLS-associated cytokines (Table 2).

**Table 2:** Correlations between sCD138 and serum cytokines

Cytokine	*R*	*p* value	N
Remission			
Angpt2	0.0667	0.88	10
VEGF	0.2667	0.49	9
CXCL10	−0.2912	0.35	12
CCL2	−0.3263	0.29	12
sTie2	−0.25	0.59	7
sVEGFR2	0.6429	0.13	7
Acute			
Angpt2	−0.6	0.35	5
VEGF	0.3367	0.33	9
CXCL10	0.233	0.55	9
CCL2	0.1667	0.677	9

### Other eGCX-related proteins in ISCLS plasma

Previously, we performed proteomic profiling of a subset of the ISCLS plasma samples included in the current study using a multiplexed aptamer‐based assay (n = 9) [15]. Further focused analysis of these results revealed a significant increase in the abundance of several eGCX-related components in acute ISCLS plasma compared to remission samples (Table 3). Glypicans (2 and 6), which are membrane heparan sulphate proteoglycans anchored by a glycosylphosphatidylinositol linkage, were increased 2.5-3-fold in acute ISCLS plasma relative to convalescent samples. Abundance of thrombospondin-1 (TSP-1), an important eCGX glycoprotein that interacts with more than 80 ligands on the endothelial cell surface including proteases, ECM components, and growth factors [26], was increased threefold in acute ISCLS plasma relative to baseline.

**Table 3:** GCX-and endothelial-related proteins in ISCLS plasma

Protein	Fold increase (acute vs. remission)	*p* value	Category
GPC2	3.06	0.0017	GCX proteoglycan
GPC6	2.57	0.009	GCX proteoglycan
TSP-1	3	0.0002	GCX proteoglycan
MMP1	2.61	0.002	Protease
MMP8	7.92	0.02	Protease
MMP9	4.54	0.0004	Protease
MMP13	1.66	0.006	Protease
MMP14	1.8	0.03	Protease
MMP17	2.45	0.001	Protease
ADAM12	2.64	0.003	Protease
ADAMTS1	3.13	0.002	Protease
ADAMTS4	2.28	0.009	Protease
ADAMTS15	2.44	0.0013	Protease
ADAMTS13	−1.06	0.69	EC-derived protease [27]
Coagulation factor III (tissue factor)	1.39	0.08	Endothelial damage
vWF	1.29	0.37	Endothelial damage [21]
sICAM1	1.18	0.15	Endothelial activation/damage [27]
VCAM1	1.13	0.24	Endothelial activation
sE-selectin	−1.04	0.63	Endothelial activation

We also detected increased quantities of eCGX-degrading enzymes including matrix melloproteinases (MMP1, 8, 8, 13, 14, 17), A Disintegrin And Metalloproteinase Domain (ADAM)12, and A Disintegrin And Metalloproteinase With Thrombospondin Motifs (ADAMTS 1, 4, 15) family members in acute compared to convalescent plasma. By contrast, amounts of ADAMTS13, a metalloprotease, which cleaves von Willebrand factor (vWF) and is secreted by activated or damaged endothelial cells [27], were not increased during ISCLS flares. Accordingly, other plasma markers of endothelial activation and/or injury, including tissue factor, vWF, soluble Intercellular Adhesion Molecule 1 (ICAM1), vascular cell adhesion molecule 1 (VCAM-1), and soluble E-selectin, were not significantly different in acute and convalescent plasma.

### eGCX components in cultured endothelial cells

**Fig. 4:**
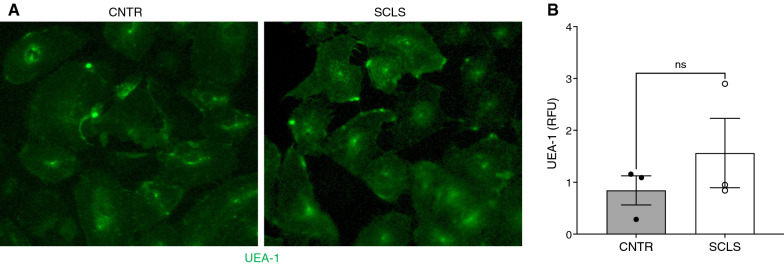
eGCX components and expression of relevant eGCX-related genes in ISCLS BOECs. **A**, **B** Representative images of fucose (**A**) in endothelial cells from subjects with ISCLS or healthy controls (n = 3/group) detected by UEA-1-FITC (green, A) and quantified (**B**). RFU = relative fluorescence units; original magnification = 10x

To explore a potential endothelial contribution to eGCX dysregulation in ISCLS, we first examined the glycocalyx layer visually in BOECs by staining with fluorescently-labeled UEA-1, a lectin that binds cell surface fucose. UEA-1 staining was similar in ISCLS and control BOECs both visually (Fig. 4A) and quantitatively (Fig. 4B), suggesting that there was no difference in eGCX content in ISCLS and control endothelial cells in tissue culture.

**Fig. 5:**
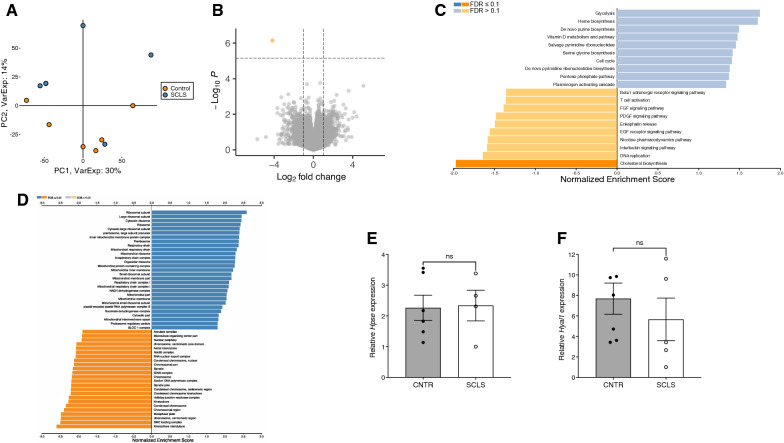
Comprehensive gene expression analysis of ISCLS BOECs.** A** Principal component analysis based on plot of PC1 and PC2 for normalized transcript counts of BOECs from ISCLS patients (n = -5, blue) and healthy controls (n = 6, orange). **B** Volcano plot of differentially expressed genes between control and ISCLS BOECs. Line represents threshold for genome-wide signficance (FDR < 0.1). The horizonal axis represents the positive (blue) and negative (orange) normalized enrichment score. **C** Gene set enrichment analysis (GSEA) of differentially expressed genes. Bar chart represents the top 20 enriched PANTHER pathway gene sets. The horizonal axis represents the positive (blue) and negative (orange) normalized enrichment score. **D** Gene set enrichment analysis (GSEA) of differentially expressed genes. Bar chart represents the significantly enriched (FDR < 0.1) gene ontology (GO) terms of the Cellular Component gene sets. **E**, **F** Relative *Hpse* (C) or *Hyal1* (D) expression in BOECs determined by quantitative real-time PCR. Values are mean ± S.E.M.; ns = not significant

Our previous results demonstrated aberrant expression of several vascular-related genes (e.g. *Eta*, encoding endothelin receptor A) in BOECs from ISCLS patients relative to control cells [16]. To evaluate the contribution of glycocalyx-related gene expression to eGCX dysfunction in BOECs, we performed RNA Seq. This analysis revealed 767 DEGs in ISCLS and healthy control BOECs (*p* < 0.05) (full list in Additional file 1: Table S1), but only one gene (CH17-260O16.1, a likely pseudogene) whose differential expression reached significance after adjustments for multiple comparisons (FDR < 0.1, Fig. 5A, B). Although these results suggested that the study was underpowered due to the limited number of available samples, Gene Set Enrichment analysis (GSEA) using the PANTHER pathway database nonetheless revealed that the cholesterol biosynthesis pathway was significantly downregulated in these cells (Fig. 5C). We further interrogated these data using a glycocalyx-related gene set obtained from Harminizome [28]. Of the glycocalyx-related genes, 60 were upregulated and 43 were downregulated in ISCLS BOECs compared to controls (Additional file 2: Table S2), although none of the DEGs reached genome-wide significance (FDR < 0.1). 3 genes including *SDC1*, which encodes CD138 (log fold change 1.89), *PROCR*, and *ANGPT2* had p-values < 0.05. GO cellular component GSEA results indicated that the several cellular compartments containing one or more glycocalyx-related genes, including organelle envelope lumen, mitochondrial matrix, vacuolar lumen, and Golgi lumen, were among the most significantly upregulated gene sets in ISCLS BOECs relative to controls (Fig. 5D and Additional file 3: Table S3). Likewise, several components with glycocalyx-related genes including chromosomal region, cell–cell junction, actin cytoskeleton, and nuclear speck were significantly downregulated in ISCLS samples relative to controls.

Finally, we evaluated expression of enzymes related to digestion of eGCX-related carbohydrates, *Hpse* (encoding heparanase) and *Hyal1* (encoding hyaluronidase), by qPCR. Expression of *Hpse* or *Hyal1* in control and ISCLS BOECs was equivalent (Fig. 5E, F), suggesting that shedding of eGCX-related components into ISCLS plasma was not due to increased expression of *Hpse* or *Hyal1*.

## Discussion

Here we present evidence for eGCX remodeling in ISCLS. Although ISCLS flares release macromolecules, including proteins up to 900 kDa, into the extravascular space, we detected increased abundance of eGCX components and eGCX-degrading enzymes in circulation in acute ISCLS relative to convalescent periods, consistent with glycocalyx shedding. eGCX dysfunction could have an impact on the severity of acute vascular leakage through several mechanisms including increasing local concentrations of proinflammatory cytokines on the endothelial surface. Microvascular hyper-permeability can be a component of critical illness due to sepsis, burns, and trauma, among others. This is reflected by increased serum levels of markers of endotheliopathy [29]. Elevated sCD138 and sTM levels predict morbidity and mortality in mechanically ventilated patients [30]. However, severe and protracted hemoconcentration and hypoalbuminemia are uncommon in these conditions [31]. Whether inflammation induces vascular hyper-permeability in critical illness and ISCLS through similar mechanisms is unknown. Indeed, ISCLS flares can even occur in the absence of overt inflammatory triggers.

Baseline sCD138 levels in ISCLS were above those typically found in healthy subjects, suggesting ongoing eGCX dysfunction in the absence of overt clinical symptoms. Thus, rather than using a single, uniform cutoff value for sCD138 in individual patients, clinicians may need to evaluate a constellation of biomarkers such as sCD138, HA, and Angpt2 in individual patients to assess episode severity and/or progression. The lack of further increase in sCD138 in acute sera in our relatively small cohort may relate specifically to the variable timing of sample collection. A subset of samples was obtained at or near the onset of acute clinical symptoms while serial measurements in several patients revealed that sCD138 levels were highest during the recovery (post-leak) phase, during which hypervolemia can occur due to mobilization of administered IV fluids from peripheral tissues. Expanded plasma volume has been previously identified as a risk factor for CD138 shedding [32]. Variability in acute sCD138 levels could also be due to the wide range of episode severity. Several samples were collected from patients whose clinical symptoms were mild and resolved quickly without hospitalization. Increased sCD138 levels may also relate specifically to the presence of MGUS. Monoclonal plasma cells produce and/or shed abnormally high amounts of sCD138, and changes in serum sCD138 levels not only correlate with progression to myeloma but also reflect therapeutic efficacy [24]. Accordingly, we observed a strong correlation between sCD138 and paraprotein levels in ISCLS.

Although the abundance of several eGCX elements (e.g. HA, sCD138) were increased in the acute phase, relative quantities of endothelial cell-derived proteins that might suggest endothelial cell injury (such as TM) were not significantly increased in active disease. Cleavage of TM by neutrophil elastase and other inflammation-related proteases leads to shedding into plasma [16]; increased sTM levels are found in critically ill patients with sepsis or trauma, and levels correlate with the extent of organ dysfunction and mortality [33]. These findings are also consistent with our previous observations that acute ISCLS sera did not induce cytotoxicity when applied to normal endothelial cells [6].

Other eGCX-related proteins may be shed into circulation during flares. GPC1 is the most well-studied glypican component of the eGCX and has been shown to protect against endothelial dysfunction related to blood vessel stiffness [34]. GPC 1, 3, and 4 are elevated in plasma of patients with sepsis and correlate with markers of disease severity, organ failure, and eGCX damage [35]. GPC1 was not measured by our array, but both GPC2 and GPC6 were elevated in acute ISCLS plasma. Although the function of GPC2 and 6 in endothelial cells has not been well studied, survey data indicate endothelial cell expression (https://www.proteinatlas.org/ENSG00000213420-GPC2/celltype and https://www.proteinatlas.org/ENSG00000183098-GPC6/celltype). ISCLS endothelial cells may exhibit aberrant patterns of GPC expression.

Increased abundance of eGCX-degrading proteases, including MMPs, ADAMs, and ADAMTS family members were detected in ISCLS plasma. These factors regulate eGCX composition during inflammation; MMP9 and 13 induce CD138 shedding by cleaving its ectodomain [36]. ISCLS-associated proinflammatory cytokines including TNFα and IL-1β, among others, increase expression of eGCX degrading proteases in endothelial cells [10]. Previously we documented widespread neutrophil degranulation in acute ISCLS [15], suggesting another cellular source of proteases. Notably, we did not visualize grossly abnormal carbohydrate content or expression of genes encoding eGCX degrading enzymes (*Hyal1, Hpse*) in cultured ISCLS endothelial cells at baseline. Thus, features of ISCLS flares not recapitulated in vitro, including increased shear stress due to hemoconcentration and increased production of reactive oxygen species (ROS) due to ischemia could adversely affect endothelial synthesis and/or degradation of eGCX elements.

Fortification of the eGCX may be a potential therapeutic approach for acute ISCLS. Low molecular weight heparin (Lovenox) is an FDA-approved anticoagulant that also reduces eGCX shedding of glycans in inflammatory states by competitively inhibiting heparanase [37]. Sulodexide, a natural mixture of GAGs including heparan sulfate and dermatan sulfate extracted from human GI tract, has shown promise in restoring eGCX components and decreasing permeability of retinal microvasculature in patients with type 2 diabetes [38].

Finally, although disease flares in our patients were prototypical, an unusual clinical aspect of one case was the finding of retinal holes or rifts. Although it is unclear whether the visual disturbance was a manifestation of active ISCLS, symptoms coincided with a period in which the patient was non-compliant with treatment and improved once terbutaline therapy was reinstated and IVIG prophylaxis was started. Visual disturbances in association with acute ISCLS flares are uncommon although isolated cases of macular edema [39] and ischemic optic neuropathy [40] have been described in case reports. Patients with ISCLS may be at increased risk for ophthalmologic complications due to hyper-coagulability associated with hemoconentration and decreased perfusion resulting from hypotensive shock [2].

## Conclusions

Circulating levels of eGCX components including sCD138 and HA might be measured to monitor the course of idiopathic ISCLS. The absence of increased levels of endothelial injury markers (e.g. thrombomodulin, tissue factor) suggests that the endothelial glycocalyx, but not the endothelium itself, damaged in active ISCLS.

## Supplementary Information

**Additional file 1.** Table S1 Differentially expressed genes (DEGs) in ISCLS and control blood-derived endothelial cells (BOECs) detected by RNA-Seq.

**Additional file 2.** Table S2 Glycocalyx-related DEGs in ISCLS and control BOECs.

**Additional file 3.** Table S3 upregulated gene sets in ISCLS BOECs relative to controls using GO cellular component Gene set enrichment analysis (GSEA).

## Data Availability

Data are available from the corresponding author on reasonable request. RNA-Seq data were deposited into GEO (Accession# GSE204882).
